# Risk-reducing salpingo-oophorectomy among Chinese women at increased risk of breast and ovarian cancer

**DOI:** 10.1186/s13048-023-01222-1

**Published:** 2023-06-29

**Authors:** Zheng Feng, Ke Zuo, Xingzhu Ju, Xiaojun Chen, Wentao Yang, Hao Wen, Lin Yu, Xiaohua Wu

**Affiliations:** 1 Department of Gynecological Oncology, Fudan University Shanghai Cancer Center, 270 Dong-an Road, Shanghai, 200032 China; 2 Department of Oncology, Shanghai Medical College, Fudan University, Shanghai, 200032 China; 3 Department of Pathology, Fudan University Shanghai Cancer Center, Shanghai, 200032 China

**Keywords:** Ovarian cancer, BRCA1, BRCA2, Risk-reducing salpingo-oophorectomy, Sectioning and extensively examining the Fimbriae

## Abstract

**Background:**

Risk-reducing salpingo-oophorectomy (RRSO) is recommended for women at increased risk of breast and ovarian cancer. We launched a prospective study of women receiving RRSO, including those with mutations in genes beyond BRCA1/2.

**Patients and methods:**

80 women were enrolled for RRSO with sectioning and extensively examining the fimbriae (SEE-FIM) protocol between October 2016 and June 2022. The majority of participants had inherited susceptibility gene mutations or a family history suggesting ovarian cancer risk, while patients with isolated metastatic high-grade serous cancer of unknown origin were also included.

**Results:**

Overall, two patients had isolated metastatic high-grade serous cancer with unknown origin, and four patients had family histories but refused to take genetic tests. The rest 74 patients harbored deleterious susceptible gene, including 43 (58.1%) with BRCA1 mutation, and 26 (35.1%) with BRCA2 mutation, respectively. Other mutated genes included ATM (1), BRIP1(1), PALB2(1), MLH1(1) and TP53 (1) in each patient. Among the 74 mutation carriers, three (4.1%) cancers were recognized, one (1.4%) was found to have serous tubal intraepithelial carcinoma (STIC), and five patients (6.8%) was diagnosed with serous tubal intraepithelial lesions (STILs). P53 signature was recognized in 24 patients (32.4%). For other genes, MLH1 mutation carrier had endometrial atypical hyperplasia and p53 signature in fallopian tubes. The germline TP53 mutation carrier had STIC in the surgical specimens. Evidence for precursor escape was also recognized in our cohort.

**Conclusion:**

Our study demonstrated clinic-pathological findings of patients at increased risk of breast and ovarian cancer, and expand the clinical application of SEE-FIM protocol.

**Supplementary Information:**

The online version contains supplementary material available at 10.1186/s13048-023-01222-1.

## Background

Ovarian cancer is one of the most common and lethal diseases among women around the world [1]. Due to the lack of effective screening strategies, over two-thirds of patients are of the advanced stage at initial diagnoses, and the five-year overall survival is around 40% [2]. Thus, effective prevention strategies are urgently required for this tough disease.

Epidemiological studies show that women with deleterious germline mutations in BRCA1 or BRCA2 gene have a higher lifetime risk of ovarian cancer [3]. In addition, other homologous recombination repair (HRR) genes and DNA mismatch repair-related (MMR) genes also show susceptibility to ovarian cancers [4, 5]. According to the NCCN guideline, BRCA1, BRCA2, BRIP1, RAD51C and RAD51D have been proved with susceptibilities of ovarian cancer, and their deleterious mutation carriers were recommended for risk reducing salpingo-oophorectomy (RRSO) [6]. Several studies have reported the management of BRCA mutation carriers, while data on other inherited genes are quite scarce [7–10].

Sectioning and extensively examining the fimbriae (SEE-FIM) protocol is performed at RRSO for the detection of occult malignancies [11]. The clinical application of SEE-FIM protocol is more extensive at our institution. Firstly, breast cancer patients always have oophorectomy as an alternative for hormonal therapy [12]. Also, opportunistic salpingectomy was recommended as a strategy for ovarian cancer prevention [13]. For breast cancer patients with other inherited genes, SEE-FIM protocol following bilateral salpingo-oophorectomy could provide more information for the genetic susceptibility to ovarian cancer. Besides, patients with suspicious pelvic mass and family histories are also recommended for SEE-FIM protocol. Furthermore, SEE-FIM protocol is also performed for isolated metastatic high-grade serous cancers to seek its origin.

We prospectively enrolled women at increased risk of breast and ovarian cancer, and performed RRSO with SEE-FIM protocol. Our study demonstrated the clinic-pathological findings of those patients, and illustrated the pathogenic features of genes beyond BRCA1/2.

## Results

### Participants

**Fig. 1:**
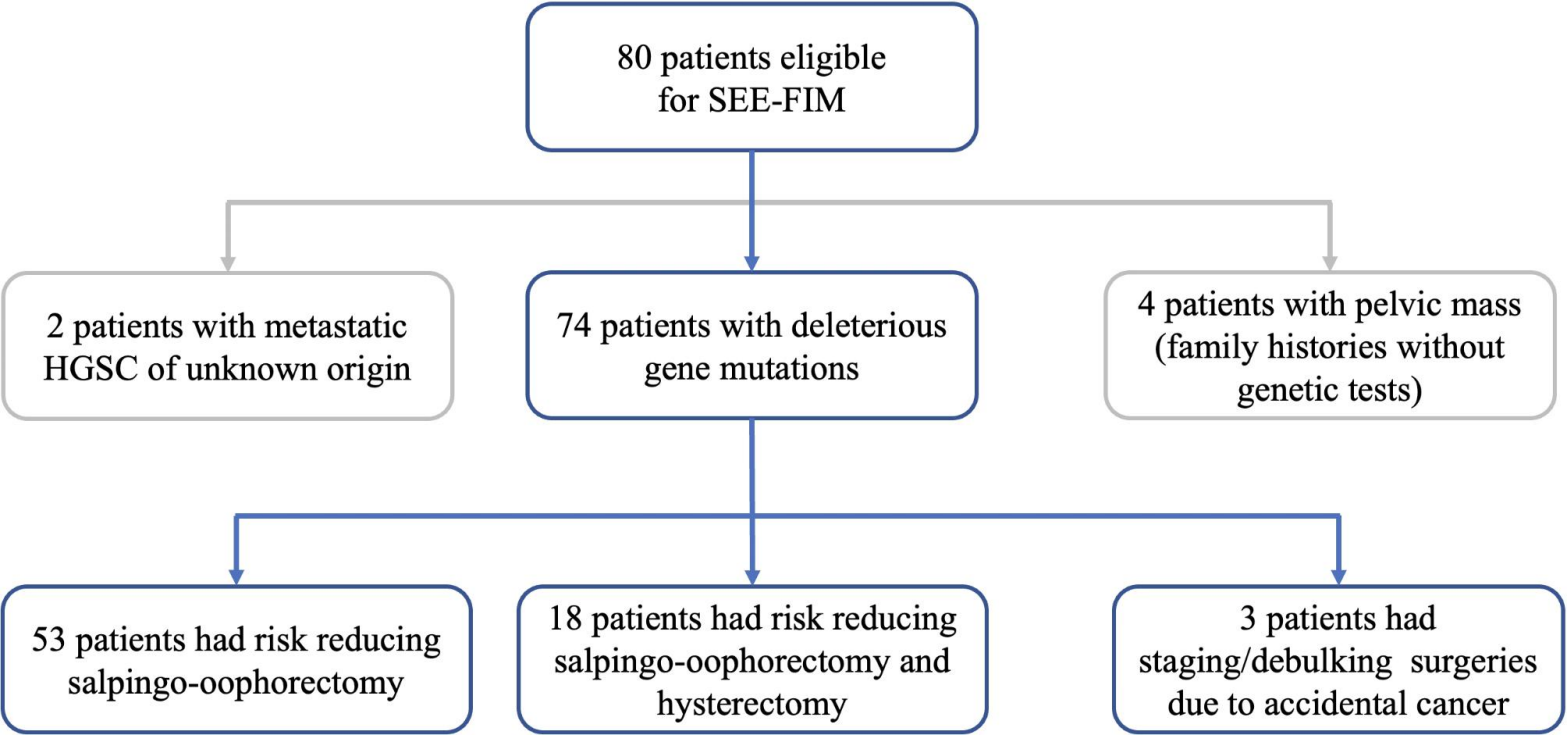
Flow diagram of patients eligible for SEE-FIM

80 women were eligible for SEE-FIM protocol (Fig. 1). Among them, two patients had isolated metastatic high-grade serous cancer with unknown origin. SEE-FIM was performed to seek for any lesion in the ovaries or fallopian tubes. Four patients had family histories but refused to take genetic tests. They were all postmenopausal and planned to have salpingo-oophorectomy due to pelvic mass. Thus, SEE-FIM was also performed. The rest 74 patients harbored deleterious susceptible gene mutations and pretended to have RRSO for further evaluation.

### Clinical characteristics

**Fig. 2:**
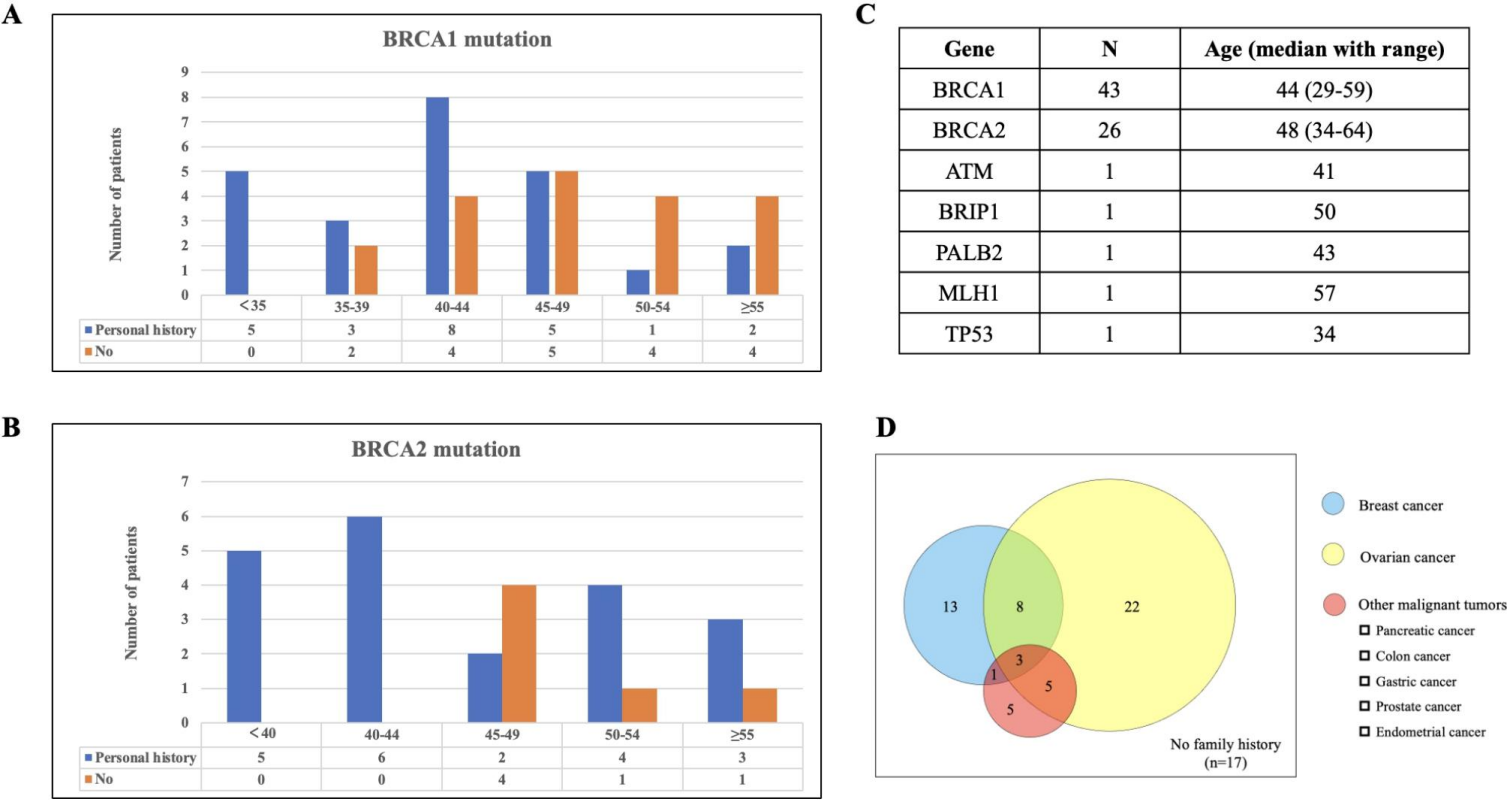
Personal and family histories of mutation carriers A, age distribution of risk-reducing surgeries in patients with BRCA1 mutation stratified by personal history B, age distribution of risk-reducing surgeries in patients with BRCA2 mutation stratified by personal history C, age distribution of risk-reducing surgeries in patients with different genes D, distribution of family histories in our cohort

Demographics of the 74 mutation carriers are provided in Table 1. The median (range) age was 46 (29–64) years old. The majority of mutation carriers had pathogenic BRCA1 and BRCA2 mutations, 43 (58.1%) with BRCA1 mutation, and 26 (35.1%) with BRCA2 mutation, respectively. Other mutated genes included ATM (1), BRIP1(1), PALB2(1), MLH1(1) and TP53 (1) in each patient. A large proportion of patients had personal history of other tumors, 45 (60.8%) with breast cancer, 1 with both gastric cancer and breast cancer, and 1 with ovarian sex cord-stromal tumor. Patients with personal history of breast cancer received RRSO at younger ages (Fig. 2A and B). Earlier intervention was performed due to concurrent genetic tests and individual requirements. 57 (77.0%) patients had family histories of related cancers (Fig. 2D). Family histories of ovarian cancer and breast cancer were most frequently reported. Other cancers including pancreatic cancer, colon cancer, gastric cancer, prostate cancer and endometrial cancer were also recognized.

**Table 1:** Patient characteristics (N = 74)

Age	median (range)	46 (29–64)	
Mutated genes	BRCA1	43	58.1%
	BRCA2	26	35.1%
	ATM	1	1.4%
	BRIP1	1	1.4%
	PALB2	1	1.4%
	MLH1	1	1.4%
	TP53	1	1.4%
Personal history	Breast cancer	45	60.8%
	Gastric cancer and breast cancer	1	1.4%
	Ovarian sex cord-stromal tumor	1	1.4%
	No	27	36.5%
Family history	Yes	57	77.0%
	No	17	23.0%
CA125	median (range)	11.0 (3.9–124)	
HE4	median (range)	45.7 (30.5–79.6)	
Surgical procedure	Staging/debulking surgery	3	4.1%
	RRSO	54	73.0%
	RRSO + hysterectomy	17	23.0%
Pathology	Cancer	3	4.1%
	STIC	1	1.4%
	STIL	5	6.8%
	p53 signature	24	32.4%
	Benign	41	55.4%

### Surgical and pathological findings

All the 74 mutation carriers pretended to had RRSO, while one patient with elevated CA125 had disseminated neoplasms, and undertook debulking surgery. Another two patients had occult malignancies (one in the fallopian tube and another in the ovary) and received subsequent staging surgery with adjuvant chemotherapy. Thus, 3 (4.1%) patients with cancers were recognized in total.

For the rest 71 patients, 54 patients had RRSO, and 17 patients had RRSO with combined hysterectomy. Five patients (6.8%) was diagnosed with STILs and one (1.4%) was found to STIC. P53 signature was recognized in 24 patients (32.4%). Patients with breast cancer were more likely to had lesions of STIL or beyond (Table S1). Details for the nine patients with precancerous or malignant lesions are listed in Table 2.

**Table 2:** List of patients with precancerous or malignant lesions

Diagnosis	Age	Gene	Personal history	Family history	CA125	HE4
Cancer	44	BRCA1	Breast cancer	Ovarian cancer	124	79.6
Cancer	49	BRCA1	Breast cancer	No	4.72	42.8
Cancer	45	BRCA2	No	No	15.7	34.75
STIC	34	TP53	Breast cancer	Breast cancer/Ovarian cancer/Pancreatic cancer	9.87	46.4
STIL	55	BRCA1	Breast cancer	Breast cancer	15.9	69.5
STIL	34	BRCA2	Breast cancer	No	13.7	46.7
STIL	38	BRCA2	Breast cancer	No	27.6	39.9
STIL	60	BRCA2	Breast cancer	No	21.5	59
STIL	50	BRIP1	No	Ovarian cancer	17.8	53.1

Until our last follow-up (July 15th, 2022), no subsequent peritoneal cancer was found among our patients. While three patients with BRCA1 mutations had other subsequent cancers. One patient suffered from both breast cancer and lung cancer. One breast cancer patient had contralateral breast cancer. Another breast cancer patient had lung cancer.

### Inherited genes beyond BRCA1/2

Details of the patients harboring other genes beyond BRCA1/2 were listed in Table 3. Although the relationship of ATM and PALB2 genes with ovarian cancer risk was not clear, RRSO was also performed in two patients considering personal and family histories. P53 signature was found and might provide benefits for ovarian cancer prevention. For Lynch syndrome associated genes, that patient had a daughter diagnosed with clear cell ovarian cancer, and deleterious MLH1 gene mutation was detected. RRSO and hysterectomy was performed, and postoperative pathology showed endometrial atypical hyperplasia and p53 signature in fallopian tubes. Another important gene should be mentioned was the oncogene TP53. A breast cancer patient harboring deleterious TP53 mutation was diagnosed with Li-Fraumeni syndrome. She underwent bilateral salpingo-oophorectomy as endocrine therapy, and STIC was identified in the surgical specimens (Figure S1).

**Table 3:** List of patients with genes beyond BRCA1/2

Gene	Age	Personal history	Family history	CA125	HE4	Surgery	Diagnosis
BRIP1	50	No	Ovarian cancer	17.8	53.1	RRSO + TAH	STIL
ATM	41	Gastric cancer/Breast cancer	Ovarian cancer/Pancreatic cancer	9.73	30.8	RRSO + TAH	p53 signature
PALB2	43	Breast cancer	Colon cancer	7.39	45.7	RRSO	p53 signature
MLH1	57	No	Ovarian cancer	12.58	53.31	RRSO + TAH	endometrial atypical hyperplasia and p53 signature in fallopian tubes
TP53	34	Breast cancer	Breast cancer/Ovarian cancer/Pancreatic cancer	9.87	46.4	RRSO	STIC

### Precursor escape from the fallopian tube

**Fig. 3:**
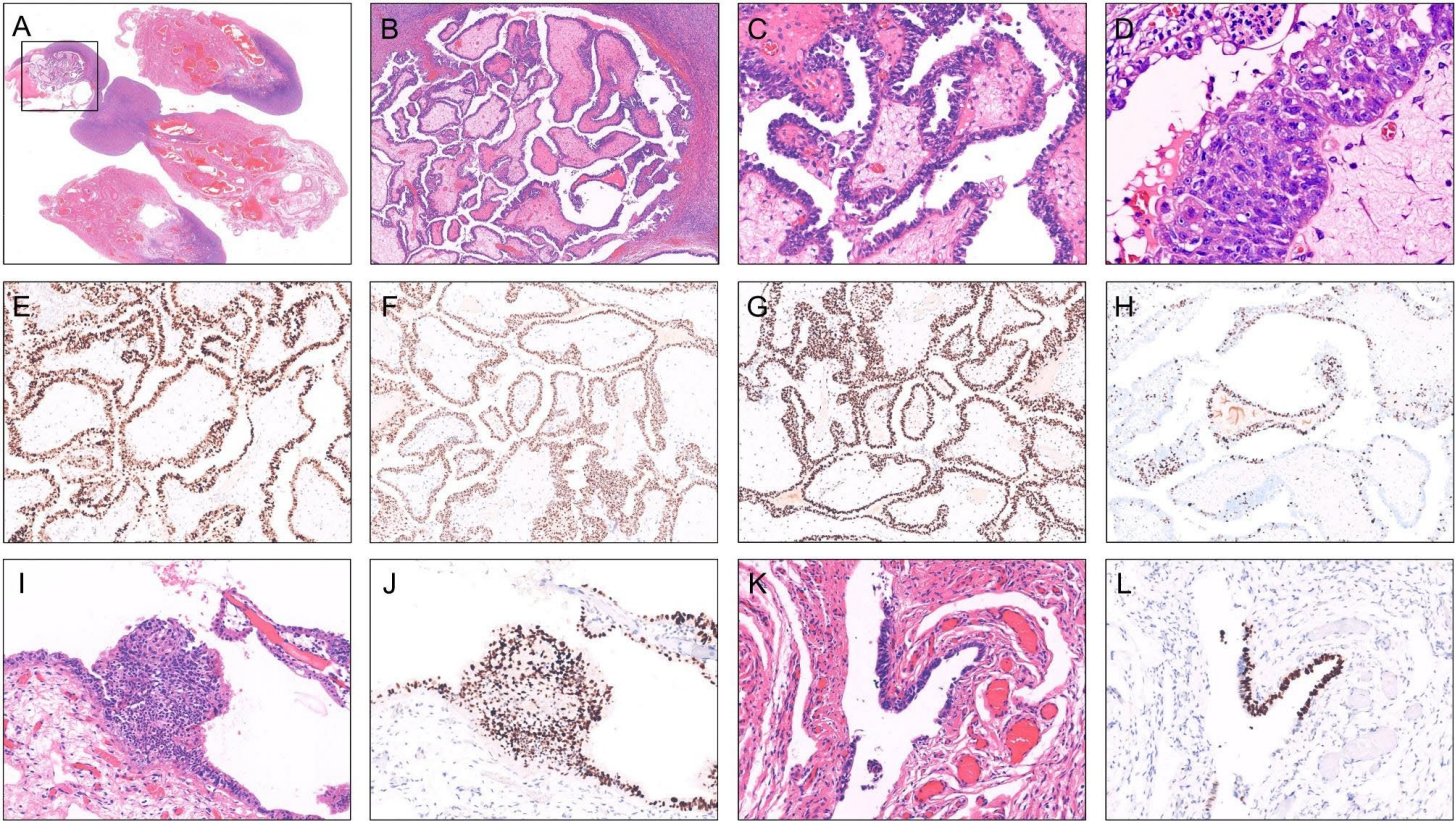
The lesions of the fallopian tube and ovary in a patient with *BRCA1* germline mutation A, a single focus of occult high grade serous carcinoma localized in the right ovary (top left quadrangle) B ~ D, the microscopic features from low power to high power. The tumor exhibited typical features of high grade serous carcinoma with papillary structures and markedly cellular atypia. Nuclei were large with nucleoli and mitoses were easily found (D) E ~ H, high grade serous carcinoma was stained immunohistochemically with p53, PAX8, WT-1 and Ki-67 respectively. This case showed strong and diffuse expression of p53 (E) and moderate-high Ki-67 index (H) I ~ J, STIC was found in the fimbriated end of the right tube (I) with strong and diffuse p53 staining (J). K ~ L, p53 signature was found in the left tube with focal strong p53 staining

One patient with occult ovarian cancer was identified in our cohort. The women had RRSO due to deleterious BRCA1 mutation. Postoperative pathology showed high-grade serous cancer in the right ovary with STIC in the right fallopian tube, while p53 signature was found in the left fallopian tube. (Fig. 3). This indicated that the origin of ovarian cancer was from the fallopian tube.

Apart from the ovary, malignancies at other sites could also be regarded. Two patients with isolated high-grade serous cancer of unknown origin had also been enrolled in our study. One had metastatic pelvic lymph nodes, and another one had metastasis on the liver surface. Genetic tests showed no germline mutations, while both tumors harbored TP53 somatic mutations. SEE-FIM was performed for the surgical specimens, and p53 signature was found in their fallopian tubes. This demonstrated precursor escape from the fimbria of the fallopian.

## Discussion

Genetic tests for ovarian cancer have been gradually applied in China, and the demand for genetic counselling and prophylactic intervention is increasing. RRSO with SEE-FIM protocol at our institution initiated accompanied with our nationwide prevalence study of germline BRCA mutation [14]. Thus, we prospectively enrolled women at increased risk of breast and ovarian cancer, and performed RRSO with SEE-FIM protocol since 2016. Our study presented the application of prophylactic surgeries in China, and demonstrated the pathogenic features of several genes beyond BRCA1/2.

The purpose of RRSO was to reduce ovarian cancer risk, and to identify disease at the precancerous period or an earlier stage. Our study found an overall 4.1% rate of cancer in mutation carriers undergoing risk-reducing surgery, including two with BRCA1 mutation and one with BRCA2 mutation. This is consistent with previous reports [7, 9, 10]. STIL and STIC were also identified in 6.8% and 1.4% of patients, respectively. However, the management of STIC is puzzled. Hoeven et al. [15] reviewed the management and outcomes of 82 BRCA1/2 carriers with isolated STIC. The estimated risk of recurrence was 11%, while no recurrences were reported in those patients who underwent staging or received chemotherapy. Patrono et al. [16] reported that 4.5% (3/67) BRCA1/2 mutation carriers with STIC developed peritoneal carcinoma. This indicated that additional treatment for STIC might reduce the recurrence risk, and intensive surveillance is necessary.

P53 signature occurred at a greater prevalence than other precursor lesions (STIC/STIL) [17, 18], and accounting for 32.4% in our cohort. However, its role in the development of ovarian cancer is not that clear. Since p53 signature can also be detected in benign specimens without pathogenic gene mutations, whether it had clinical implication remains unveiled. Akahane et al. [19] compared TP53 variants of p53 signatures between specimens of RRSO and benign controls. Larger proportion of p53 signatures in BRCA1/2 mutation carriers harbored pathogenic TP53 variants. While p53 signatures in the control group did not had deleterious mutations. Another experimental study showed that double-knockout of TP53 and BRCA1 in the mouse fallopian tube-derived organoids led to tumor formation [20]. Thus, p53 signature should be managed with cautions, and subsequent surveillance could not be avoid.

The average ages of surgery for mutation carriers in our cohort were older than the ages recommended by NCCN guidelines [6]. The main reason could be due to the ages of the women at the time they underwent genetic testing. Patients with personal history of breast cancer received RRSO at younger ages, even earlier than ages recommended by NCCN guidelines. Those patients might have demand for hormonal therapy, or they were more anxious about cancer susceptibility.

Different from previous reports, over half of women in our cohort had personal history of breast cancer. Lee et al. [8] have evaluated pathologic findings at RRSO in BRCA mutation carriers with breast cancer. All occult invasive cancer cases were detected in patients older than 40 years, and precursor lesions in BRCA2 mutation carriers were only detected in those older than 40 years. In our cohort, all the occult cancer cases were also detected in patients older than 40 years. However, two BRCA2 mutation carriers with precursor lesions were younger than 40, while the recommended age of RRSO for BRCA2 mutation carriers were 40 to 45 years. Occult cancer might develop several years since the onset of precursor lesion. RRSO should be managed individually, considering personal histories, family histories and the detailed variants of each gene.

Interestingly, our study also reported RRSO for inherited genes beyond BRCA1/2, including other HRR genes, Lynch syndrome associated genes, and TP53 gene (Li-Fraumeni syndrome). All cases in our cohort had p53 signature or precancerous lesions. Data for these genes were quite scarce according to literature [21–23]. Whether these lesions occurred accidentally or genetically need further investigation with larger sample size.

The fundamental mechanism for SEE-FIM protocol at RRSO was the fallopian tubal theory of high-grade serous carcinogenesis [24]. Synchronous fallopian precursor lesion and occult high grade serous ovarian cancer was detected in our cohort. Moreover, SEE-FIM was also performed for isolated metastatic high grade serous cancer with unknown origin. P53 signature indicated much earlier precursor escape from the fallopian tubes.

Although our sample size was relatively small, especially for mutation carriers of genes beyond BRCA1/2. To our knowledge, this is the largest single institution study of RRSO in China, and elucidated the development of genetic counseling and prophylactic surgeries.

Our study had several strengths. One strength is the prospective design and our single institutional study could provide consistent genetic counselling, and subsequent pathologic and surgical protocols. Besides, our study expanded the application of SEE-FIM protocol. Furthermore, our study provided valuable data for other inherited genes, which was quite scarce and need further investigations.

In conclusion, our prospective study demonstrated clinic-pathological findings of patients at increased risk of breast and ovarian cancer, and expanded the clinical application of SEE-FIM protocol.

## Methods

### Participants

This prospective, observational study was conducted at Fudan University Shanghai Cancer Center. Women consented to be included in an IRB approved tissue bank prior to surgical intervention (050432-4-1212B).

80 women were enrolled for RRSO with SEE-FIM protocol between October 2016 and June 2022. The inclusion criteria were as follows: (1) known pathogenic germline mutation of breast and ovarian cancer susceptibility, (2) pelvic mass in combination with a family history of ovarian cancer, (3) isolated metastatic high-grade serous cancer of unknown origin. In our study, breast and ovarian cancer susceptibility included BRCA1, BRCA2, other homologous recombination DNA repair (HRR) genes, mismatch repair (MMR) genes and TP53 gene. Patient characteristics including age, personal and familial history, surgical procedures, pathology reports, and postoperative follow-up and so on were prospectively collected.

### Pathologic examination of tubes and ovaries

All salpingo-oophorectomy specimens were entirely submitted for histological analysis. The tube and ovary was sectioned at 2- to 3-mm intervals and the fimbriated end were extensively sectioned by the SEE-FIM protocol [25]. All sections of tissue blocks from the fallopian tubes and ovaries were stained for hematoxylin and eosin (H&E). Additional immunohistochemical staining for p53 and Ki-67 was performed for each tissue block of fallopian tubes as well. Slides were reviewed for occult invasive carcinoma (e.g. high grade serous carcinoma), serous tubal intraepithelial carcinoma (STIC), serous tubal intraepithelial lesions (STIL) and p53 signature by two gynecologic pathologists. If the review was discordant, additional review was conducted by a third pathologist. The diagnostic criteria were based on a previously validated algorithm [26, 27].

### Statistical analyses

SPSS statistical software (version 21.0, SPSS, IBM Inc, New York, USA) was used for the statistical analyses. Descriptive statistics were used for the demographic data and are summarized as the medians with the ranges, or the frequencies with the percentages. The categorical data were compared with chi-square tests. P < 0.05 was considered statistically significant, and all reported P values were 2-sided.

## Electronic supplementary material

Below is the link to the electronic supplementary material.

Supplementary Material 1: The lesions of the fallopian tube in a patient with *TP53* germline mutation.
A, a focus of STIC showed suddenly increased cellular atypia compared with the surrounding mucosa. The nuclear of STIC was enlarged and varied in shape
B, strong p53 staining occurred multifocally in the entire tube. Except the focus of STIC, the rest mucosa with strong p53 expression meet the criteria of STIL with less atypia and lower Ki-67 index

Supplementary Material 2: Pathologic findings stratefied by personal history

## Data Availability

The institutional database contains sensitive patient information, which is available upon request. Anyone who is interested in this information should contact the corresponding authors at wu.xh@fudan.edu.cn.

## Abbreviations

RRSO: Sectioning and extensively examining the fimbriae
SEE-FIM: Sectioning and extensively examining the fimbriae
HRR: homologous recombination repair
MMR: mismatch repair-related
STIC: serous tubal intraepithelial carcinoma
STIL: serous tubal intraepithelial lesions
