# Supplementary figures and images for: Risk-reducing salpingo-oophorectomy among Chinese women at increased risk of breast and ovarian cancer
Source: J Ovarian Res. 2023 Jun 29;16:125. doi: 10.1186/s13048-023-01222-1 (PMC10308750; doi:10.1186/s13048-023-01222-1)

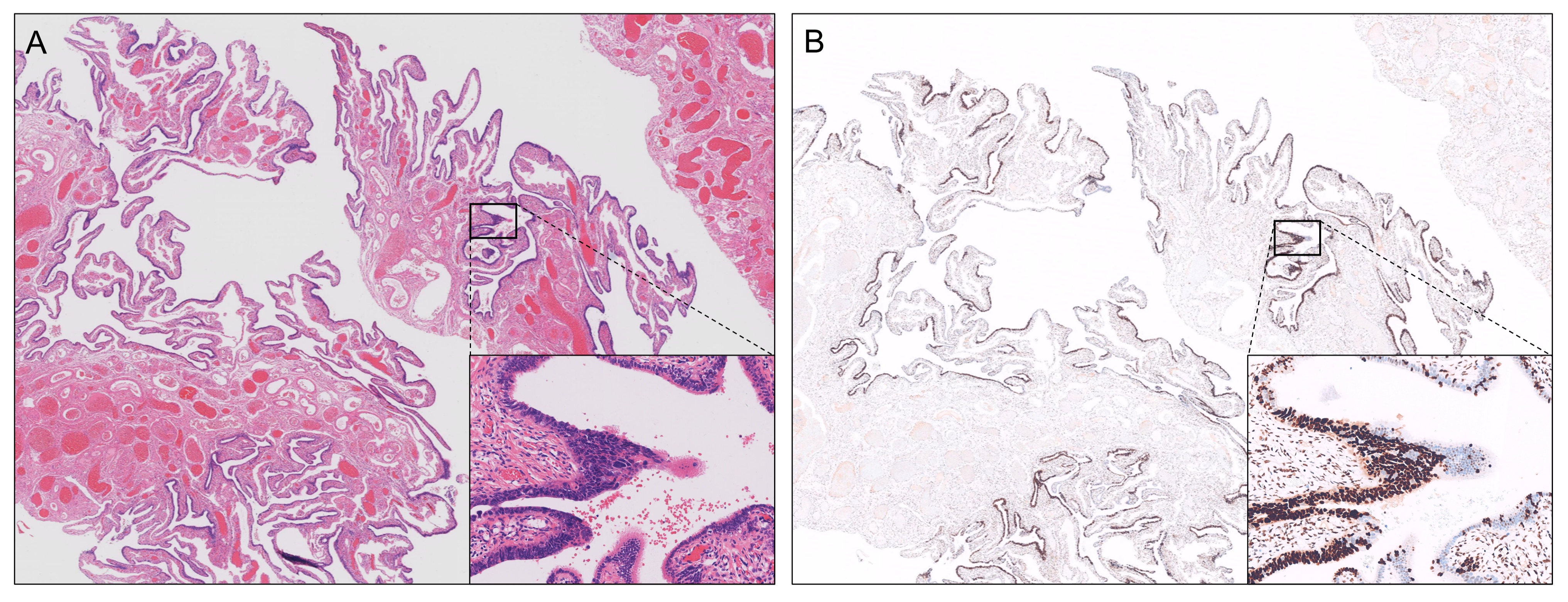

Supplement: Supplementary file 1 — Supplementary Material 1: The lesions of the fallopian tube in a patient with TP53 germline mutation. A, a focus of STIC showed suddenly increased cellular atypia compared with the surrounding mucosa. The nuclear of STIC was enlarged and varied in shape B, strong p53 staining occurred multifocally in the entire tube. Except the focus of STIC, the rest mucosa with strong p53 expression meet the criteria of STIL with less atypia and lower Ki-67 index [file 13048_2023_1222_MOESM1_ESM.jpg]
