# Supplementary material for: Risk-reducing salpingo-oophorectomy among Chinese women at increased risk of breast and ovarian cancer
Source: J Ovarian Res. 2023 Jun 29;16:125. doi: 10.1186/s13048-023-01222-1 (PMC10308750; doi:10.1186/s13048-023-01222-1)
Supplement: Supplementary file 2 — Supplementary Material 2: Pathologic findings stratefied by personal history [file 13048_2023_1222_MOESM2_ESM.docx]

| **Table S1. Pathologic findings stratefied by personal history** | | | | |
| --- | --- | --- | --- | --- |
| **Personal history of breast cancer** | | **Yes** | **No** | **P value** |
| **Pathology** | Benign | 24 | 17 | 0.559 |
|  |  | 52.20% | 60.70% |  |
|  | P53 signature | 15 | 9 |  |
|  |  | 32.60% | 32.10% |  |
|  | STIL/STIC/Cancer | 7 | 2 |  |
|  |  | 15.20% | 7.10% |  |
